# ALBACOVIDIOL Study: Effect of Calcifediol Treatment on Mortality in Patients Hospitalized for COVID-19: A Retrospective Analysis

**DOI:** 10.3390/nu17121968

**Published:** 2025-06-10

**Authors:** José Antonio Blázquez-Cabrera, Javier Torres-Hernández, Roger Bouillon, Antonio Casado-Díaz, José Manuel Quesada-Gomez, Laura Navarro-Casado

**Affiliations:** 1 Internal Medicine Department, Complejo Hospitalario Universitario de Albacete, 02008 Albacete, Spain; joseantonio.blazquez@uclm.es; 2 Clinical Biochemistry Department, Complejo Hospitalario Universitario de Albacete, 02008 Albacete, Spain; jthernandez@sescam.jccm.es (J.T.-H.); lnavarro@sescam.jccm.es (L.N.-C.); 3 Clinical and Experimental Endocrinology, Department of Chronic Diseases and Metabolism, KU Leuven, Herestraat, 3000 Leuven, Belgium; 4 Unidad de Gestión Clínica de Endocrinología y Nutrición-GC17, Instituto Maimónides de Investigación Biomédica de Córdoba (IMIBIC), Hospital Universitario Reina Sofía, 14004 Córdoba, Spain; bb1cadia@uco.es (A.C.-D.); md1qugoj@uco.es (J.M.Q.-G.); 5 CIBER de Fragilidad y Envejecimiento Saludable (CIBERFES), 14004 Córdoba, Spain; 6 Departamento de Enfermería, Farmacología y Fisioterapia, Universidad de Córdoba, 14004 Córdoba, Spain

**Keywords:** baricitinib, calcifediol, calcitriol, corticoids, COVID-19, dexamethasone, prednisone, respiratory distress syndrome (ARDS), SARS-CoV-2, VDR

## Abstract

**Background:** Immunomodulatory treatments targeting excessive host immune responses favorably shifting the course of COVID-19. High doses of calcifediol may reduce the mortality of this infection. **Objective:** To evaluate how a high dose of calcifediol modifies the risk of death in patients hospitalized with COVID-19 during the first outbreaks. **Design:** A retrospective, observational study to evaluate the relationship between treatment with calcifediol and the risk of death in patients hospitalized with COVID-19 at the “Complejo Hospitalario Universitario de Albacete” (CHUA), Spain, during the months of January to March 2021. Patients were treated with corticosteroids, and some patients also received baricitinib and/or high doses of calcifediol, according to CHUA’s therapeutic protocol 2021 for COVID-19. The primary outcome measure was mortality according to calcifediol treatment. **Results:** A total of 230 patients were included. 25(OH)D levels were measured on admission in 148 patients, showing a high prevalence of vitamin D deficiency [median 25(OH)D: 17.5 ng/mL]. Thirty-four (23%) had severe deficiency (25(OH)D ≤ 10 ng/mL). In the 119 patients (51.7%) who received in-hospital treatment with a high dose of calcifediol, the mortality rate was 12.6% (15 cases, 95% confidence interval [CI], 7.8–19.8%), while in 111 patients who did not receive treatment with calcifediol, the death rate was 23.4% (26 cases, 95% CI: 16.5–32.1%; *p* = 0.039). The odds ratio (OR) in treated vs. untreated patients was 0.47 (95% CI: 0.23–0.95). Among the patients admitted with severe deficiency, 16 received treatment with calcifediol, with a mortality rate of 0.0% (0 cases, 95% CI: 0.0–19.4%), while in the 18 not treated with calcifediol, a death rate of 38.9% was observed (7 cases, 95% CI: 20.3–61.4%; *p* = 0.008). The mortality rate was lower in patients treated with the combination of calcifediol and corticosteroids vs. those treated with corticosteroids alone (*p* = 0.038) and vs. those treated with corticosteroids and baricitinib (*p* = 0.033). **Conclusions:** In the ALBACOVIDIOL study, calcifediol treatment was associated with a lower observed mortality rate in hospitalized patients with COVID-19 treated with corticosteroids (with or without baricitinib), especially in those with severe vitamin D deficiency. Causality cannot be inferred due to the retrospective study design. (Public database: ClinicalTrials.gov, NCT05819918).

## 1. Introduction

The pandemic of coronavirus disease 2019 (COVID-19), caused by the SARS-CoV-2 β-coronavirus, is one of the most lethal in human history [1] and one of the greatest challenges that modern medicine and public health systems have faced [2]. With great acute morbidity, prolonged COVID-19 affects multiple body systems and persist for months, with severe functional organ impairment. In early outbreaks, it had a devastating impact on national economies and remains an active area of research worldwide [1].

Since the beginning of the pandemic, it has been known that the pathophysiological, immunological and clinical spectrum of COVID-19 is variable [3]. Most patients have an adequate immune response, usually of short duration, which is sufficient to eliminate the virus, after which the immune response declines and the patient recovers [4]. Approximately 20% had severe symptoms, of whom 5% had acute respiratory distress syndrome (ARDS) and often multi-organ failure, accompanied by a high risk of death [5].

SARS-CoV-2 infection, in addition to direct cellular damage [6], may also induce dysfunctional immune responses, resulting from an unbalanced host response to reduced innate antiviral defenses [7], with exuberant production of cytokines and chemokines in the so-called ‘cytokine storm’ [8,9,10] and activation of the renin–angiotensin–aldosterone system (RAAS), with decreased angiotensin-converting enzyme 2 (ACE2) [11]. These responses result in severe, aggressive, and uncontrolled inflammatory status, which is instrumental in the progression to more severe stages of the disease. COVID-19 usually begins in and predominantly affects the lungs, leading to ARDS, with multiple extrapulmonary manifestations [12].

At the beginning of the pandemic, there were no vaccines, specific antiviral treatments, or validated treatments for the disease. In this scenario, treatment of patients hospitalized with COVID-19 required adequate simultaneous management of oxygenation and inflammation, without compromising viral clearance [3]. Therefore, repurposing of systemic anti-inflammatory and immunosuppressive drugs such as glucocorticoids (dexamethasone, hydrocortisone, prednisolone, and methylprednisolone) [13], Janus kinase (JAK) inhibitors such as baricitinib and tofacitinib, and cytokine antagonists (targeting interleukin-6 receptor: tocilizumab, sarilumab; interleukin-1 receptor: anakinra; tumor necrosis factor-α: infliximab) was proposed for patients hospitalized with COVID-19 [14].

Currently, unlike SARS-CoV, which is now mitigated and MERS-CoV, which has remained geographically restricted, SARS-CoV-2 has spread globally with a high and rapid mutation rate. The viral evolution of SARS-CoV-2, with the emergence of new variants with increased infectious potential, is a cause for concern [15] and requires revisiting of the strategy employed at the beginning of the pandemic for the use of safe drugs, approved for another indication and repositioned to improve symptoms and clinical outcomes in patients with COVID-19 [14].

Corticosteroids are anti-inflammatory and immunosuppressive drugs used for the treatment of autoimmune and inflammatory diseases and in cases of cytokine storms because of their effectiveness and potency [16]. In the early outbreaks of the pandemic, they were formally contraindicated and only used in exceptional cases and after a reasoned clinical decision [17]. After the publication of the RECOVERY trial [13], corticosteroid therapy became, in clinical practice guidelines worldwide, the treatment of choice in patients hospitalized with COVID-19 and requiring supplemental oxygen [18]. However, the use of corticosteroids may compromise viral clearance and increase mortality in patients treated early in the disease [19,20]. In addition, corticosteroids can produce serious adverse effects due to interference with the action of other drugs, coagulation, and carbohydrate, protein, and lipid metabolism, and increase the risk of serious, rare infections [21].

Selective and reversible inhibition of Janus kinase (JAK) 1/2 signalling, especially with baricitinib, can act on COVID-19 through different mechanisms: anti-SARS-2 activity, inhibiting viral entry and replication, inhibition of insensitivity-associated kinases (NAKs), preventing type 1 interferon-mediated increase in angiotensin-2 converting enzyme, reducing viral endocytosis, and impairment of adaptor protein kinase 1–associated protein kinase 1 (AP) [22,23], by acting on inflammatory pathways that modulate the signalling pathway [24], preventing the activation of signal transducers and activators of cytokine transcription (IL-2, IL-6, IL-10, GM-CSF, and INF-γ), decreasing the risk of a cytokine storm. Therefore, baricitinib, alone or in combination with corticosteroids, could be considered beneficial in the treatment of COVID-19 [18].

In the early months of the pandemic, it was proposed that activation of the vitamin D endocrine system (VDES) could be useful in the treatment of COVID-19, by multiple mechanisms [25]: enhancement of innate antiviral effector mechanisms through induction of antimicrobial peptides and autophagy [26]; mitigation of the host reactive hyperinflammatory phase by decreasing the cytokine/chemokine storm; modulation of RAAS expression and neutrophil activity, helping to maintain the integrity of the pulmonary and intestinal epithelial barrier and stimulating epithelial repair; and decreasing, by direct and indirect mechanisms, the hypercoagulability and prothrombotic tendency associated with severe COVID-19 progression [27]. The use of relatively high doses of calcifediol, in the pilot clinical trial [28] and other observational intervention studies, dramatically decreased disease severity, reducing the need for ICU admission and/or mortality [29,30].

The aim of the present observational study was to evaluate how the use of calcifediol in real medical practice modifies the risk of death of hospitalized patients with COVID-19 in the Hospital General Universitario de Albacete during the first outbreaks of the pandemic, before the start of vaccinations.

## 2. Patients and Methods

### 2.1. Overview of the Study

This is a retrospective, observational, anonymized study to evaluate the relationship between treatment with calcifediol and the risk of death in patients hospitalized for COVID-19. The retrospective analysis was approved (12 May 2021) by the Biomedical Research Ethics Committee of the Complejo Hospitalario Universitario de Albacete [committee reference number 2021 36 (Eoms)] and registered in the public database ClinicalTrials.gov (NCT06279910). Conducted from MXXI medical records v. 5.0 (Selene, Siemens Healthineers, Munich, Germany), Laboratory Information System (Omega 3000 3.3 (Roche Diagnostics, Spain) and pharmacy files in patients admitted to the “Complejo Hospitalario Universitario Albacete” (CHUA), Spain, by COVID-19 (Appendix A). Hospitalized patients were included, from 24 January 2021 to 8 March 2021. Patients were followed up during admission until discharge. All patients were treated according to the hospital protocol, following the best available care treatment for COVID-19 and associated comorbidities at that time (therapeutic protocol v. 11.0, January 2021, for COVID-19 of the CHUA). The COVID-19 in-hospital treatment protocol basically included corticosteroid treatment (dexamethasone 6 mg or prednisone 40 mg or 6-methylprednisolone 30 mg/day), starting on the fifth day of disease progression, as the anti-inflammatory and immunomodulator of choice, and/or optionally baricitinib (4 mg/day starting on the fifth day of onset of disease symptoms, for 10–14 days), and high-dose calcifediol. The decision to treat with calcifediol and/or other available agents was at the judgment of each specialist in charge of the patient in the real-world scenario during the pandemic in which the treatment was proposed. For the present analysis, patients were assigned to one of two treatment groups. Group (1): oral calcifediol (25-hydroxyvitamin D 3) in drinkable ampoules at a dose of 0.532 mg, then 0.266 mg on days 3, 7, 14, 21, and 28 [28]; Group (2): not treated with calcifediol.

Inclusion criteria: (1) Patients admitted to the CHUA, (2) who meet the clinical criteria of COVID-19 (respiratory infection confirmed by radiographic pattern of viral pneumonia), (3) confirmed by a positive antigen detection test or polymerase chain reaction, and (4) who have completed, according to protocol, treatment with calcifediol. Exclusion criteria: (1) Patients who have not completed the calcifediol treatment according to protocol. Adequate calcifediol treatment was established as one loading dose of 0.532 mg + 1 or/and more additional doses of 0.266 mg; (2) patients for whom data from electronic medical records cannot be collected; (3) patients with other serious concurrent diseases (e.g., advanced oncological pathology).

The study was conducted in accordance with the Declaration of Helsinki and current Spanish legislation (Royal Decree 223/2004 on clinical trials, Law 14/2007 on biomedical research, and Organic Law 3/2018, of 5 December, on Personal Data Protection and guarantee of digital rights), as well as with the recommendations for good clinical practice. Overall, the following principles were assumed: (1) Anonymity of the information provided by the participants; (2) restriction of the data collected exclusively to the proposed study; (3) the researchers maintained the anonymity of the participants; and (4) documents were kept in secure custody, accessible only to the research team and/or authorized personnel.

Measurement of 25(OH)D was done by HPLC (Recipe), HP1260-Agilent kit, Agilent Technologies, Inc., Headquarters Santa Clara, CA, USA (intra- and inter-assay coefficient of variation at various serum concentrations < 10%).

Primary outcome measures: mortality, differentiating calcifediol treatment groups. Secondary outcomes: (1) mortality in relation to admission 25(OH)D levels; (2) to assess the effect of calcifediol treatment on mortality in patients with severe 25(OH)D deficiency.

The STROBE (Strengthening the reporting of observational studies in epidemiology) checklist was followed in the preparation of this report.

### 2.2. Statistical Analysis

All analyses were performed using R Statistical Software (v4.0.2; R Core Team 2022). An alpha level of 0.05 was established for statistical tests. As this is a retrospective study, we did not analyze the results on an intention-to-treat basis (in contrast with such method for controlled studies). A descriptive analysis was performed for all study variables, including demographic (age, sex...), clinical (comorbidities, outpatient treatment, O_2_ saturation on admission, death...), and analytical parameters of the hospital COVID-19 profile, and serum 25(OH)D levels, individually segregated according to the factors relevant to the study. Characterization included data distribution, central tendency (mean, median...) and dispersion (standard deviation, interquartile range...). Comparisons between groups were performed using statistical tests (parametric or non-parametric) appropriate to each situation and data characteristics (including, but not limited to, t-student, U-Mann–Whitney, Chi-square...). Confidence intervals were calculated using Bootstrap methodology by the Bias Corrected and Accelerated (BcA) method. The normality of the data was verified using the Lilliefors test. Odds ratio (OR) was calculated using the Odds Ratio function (DescTools package, Wald’s method) on the different contingency tables [31,32]. Univariate and multivariate logistic analyses were performed using the Firth penalized likelihood method (logist package) to mitigate analytical bias due to the small sample size and complete separation situations (observed in some subgroups). First, a univariate analysis was performed, selecting the most important clinical and therapeutic variables. Second, multivariate logistic regression was performed with the variables statistically significant in the univariate analysis.

## 3. Results

### 3.1. Patient Characteristics

**Figure 1:**
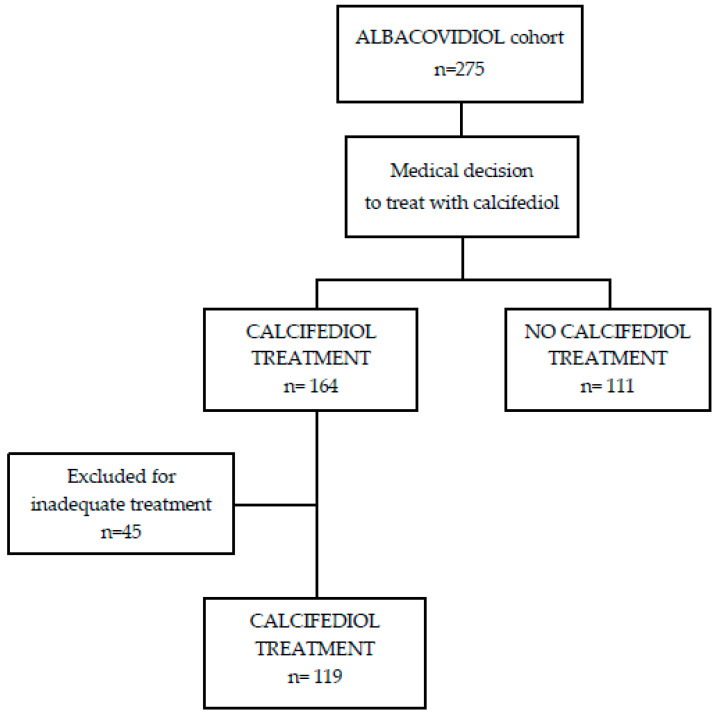
Flow chart of the patients included in the study.

From an initial population of 275 patients, medical treatment with calcifediol was initiated in 164, compared to 111 without treatment. Subsequently, from the treatment group, 45 patients were excluded because it was found that they had not received the established minimum dose of calcifediol due to absence of the initial or subsequent dose (inadequate treatment group). The final number of patients included was 230 (Figure 1 and Table S1).

At the time of admission, there was no difference between calcifediol-treated and untreated patients in relation to age, sex, and comorbidities. There was a difference in serum ferritin and D-dimer (Table 1). Of the 230 patients included in the study, serum 25(OH)D levels at admission were measured in 148 (64.3%). Of these 84 were male (56.8%) and 64 female (43.2%). The overall median 25(OH)D was 17.5 ng/mL (17.2 ng/mL in the calcifediol treatment group and 17.8 ng/mL in the untreated group). Those who eventually died during hospitalization on admission had a median serum 25(OH)D of 11.7 ng/mL vs. 18.8 ng/mL for those who did not die (ns). There was no difference between groups in in-hospital treatment with corticosteroids, but there was a difference with baricitinib (68.0% in treated vs. 45% in untreated, *p* < 0.001) (Table 1).

### 3.2. Pre-Admission Treatment with Vitamin D Metabolites: 25(OH)D Levels and Severity Parameters

Prior to hospital admission, 65 patients (28.3%) were receiving treatment with ‘vitamin’ D for other health goals: calcifediol [25(OH)D_3_] 44 (19.1%), cholecalciferol (vitamin D_3_) 24 (10.4%), and 3 were prescribed both metabolites (1.3%). No previous treatment was prescribed for 165 patients (71.7%) (Table 2). In pre-treated patients at admission, 25(OH)D levels were higher [22.2 ng/mL (median 18.9 ng/mL)] than in untreated patients [15.2 ng/mL (median 13.1 ng/mL)] (*p* < 0.001). On admission, we observed no difference in severity parameters. Forty-one patients (17.8%) died during admission (26 men and 15 women). There were more deaths among the untreated patients (ns) (Table 2).

On admission, of the 148 patients who had 25(OH)D measured, 34 patients (23%) had severe 25(OH)D deficiency (≤10 ng/mL) (Table 3).

### 3.3. Effect of In-Hospital Treatment with Calcifediol on Mortality

**Figure 2:**
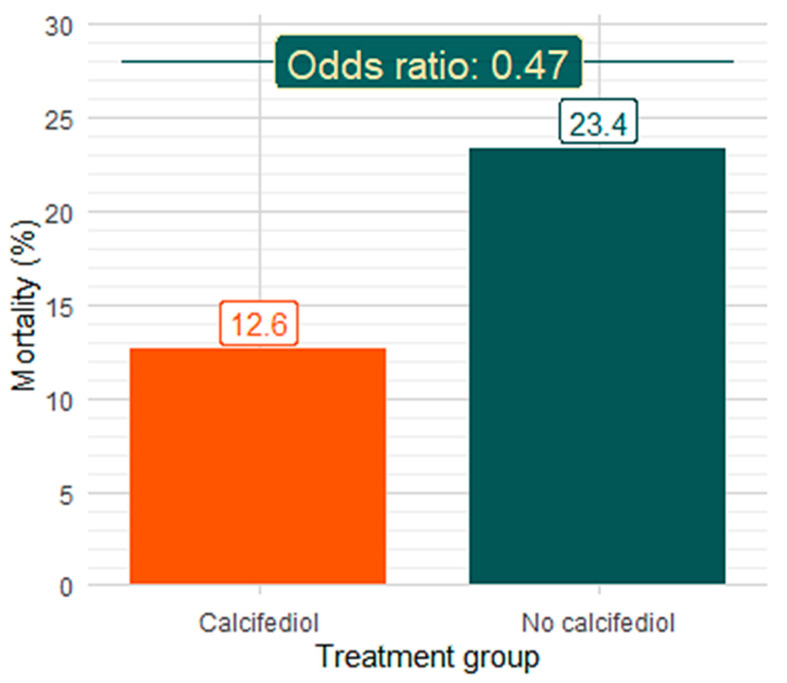
Mortality according to in-hospital treatment with high-dose calcifediol (orange) or without calcifediol (green).

**Figure 3:**
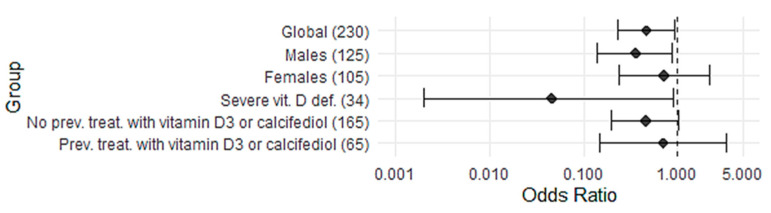
Odds ratio of mortality according to treatment with or without calcifediol.

In the 119 patients (51.7%) who received in-hospital treatment with the adequate dosage of calcifediol, the death rate was 12.6% (15 cases, 95% CI: 7.8–19.8%), while in the 111 patients who did not receive calcifediol treatment, a death rate of 23.4% (26 cases, 95% CI: 16.5–32.1) was observed. The odds ratio (OR) in treated vs. untreated was 0.47 (95% CI: 0.23–0.95), *p* = 0.039,(Figure 2 and Figure 3, Table 2 and Table 4). Multivariate logistic regression on mortality showed the following results. For the overall population (230 patients): calcifediol treatment, OR 0.476 (95% CI, 0.218–1.010), *p* = 0.053; age, OR 1.08 (95% CI, 1.05–1.13), *p* < 0.001; SpO_2_/FiO_2_, OR 0.993 (95% CI, 0.989–0.997). For males (125 patients): calcifediol treatment, OR 0.23 (95% CI, 0.08–0.63), *p* = 0.002; age, OR 1.09 (95% CI, 1.05–1.15), *p* < 0.001; SpO_2_/FiO_2_, OR 0.994 (95% CI, 0.988–1.000), *p* = 0.003 (Tables S2–S5).

In the analysis of mortality by sex, out of a total of 125 males, we observed that in the 63 who received treatment, mortality was 12.7% (8 of the 63, 95% CI, 6.6–23.1), while in the 62 who did not receive treatment, mortality was 29.0% (18 of 62, 95% CI, 19.2–41.3). The OR was 0.36 (95% CI: 0.14–0.89), *p* = 0.029. In the 105 women, no statistically significant differences were observed, with 12.5% (7 of 56, 95% CI, 6.2–23.6) treated deaths and 16.3% (8 of 49, 95% CI, 8.5–29.0%) untreated deaths (OR = 0.732, 95% CI, 0.24–2.19), *p* = 0.590 (Figure 3, Table 4).

### 3.4. Effect of Calcifediol Treatment on Mortality According to Severe 25(OH)D Deficiency

Of the 148 patients with baseline 25(OH)D levels measured on admission, 34 (23%) were severely deficient [25(OH)D ≤ 10 ng/mL, Table 3], of whom 16 were treated with calcifediol, with a death rate of 0.0% (0 cases, 95% CI, 0.0–19.4), while in the 18 who did not receive calcifediol treatment, a death rate of 38.9% (7 cases, 95% CI, 20.3–61.4, *p* = 0.008) was observed, with OR = 0.046 (95% CI, 0.0032–0.897) (Figure 3, Table 4). In the univariate analysis, only treatment with calcifediol was significant: OR 0.05, CI 95%, <0.001–0.045; *p* = 0.004 (Table S6).

### 3.5. Effect of Calcifediol on Mortality According to Baricitinib Treatment

As main immunomodulatory treatments, corticosteroids were used in virtually 100% of patients, and baricitinib in 131 patients (57). Of these, 81 (61.8%) received calcifediol, with a mortality of 17.3% [95% CI, 0.6–26.9], compared to 24% [95% CI, 14.3–37.4] of the 50 (38.2%) who did not receive calcifediol. Ninety-eight patients (42.6%) were not treated with baricitinib. Of these, 38 (38.8%) received calcifediol, with a mortality of 2.6% [95% CI, 0.5–13.5], compared to 23.3% [95% CI, 14.4–35.4] of the 60 (61.2%) who did not receive calcifediol.

**Figure 4:**
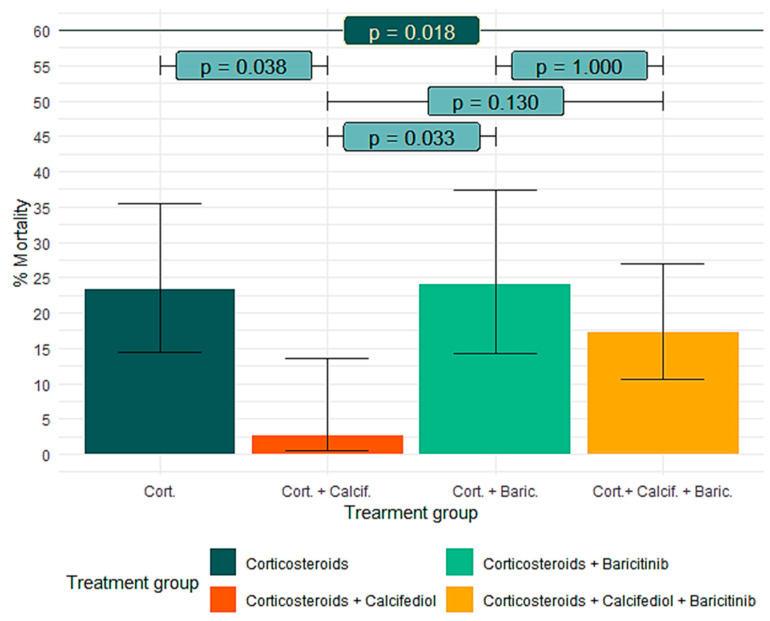
Treatment groups and mortality. Comparison between groups showed significant differences in mortality ratio (*p* = 0.018).

Comparison between groups showed significant differences in mortality ratio (*p* = 0.018) (Table 5). The mortality rate was significantly lower in patients treated with the combination of calcifediol and corticosteroids vs. patients treated with corticosteroids alone (*p* = 0.038) and vs. patients treated with corticosteroids and baricitinib (*p* = 0.033) (Table 5, Figure 4).

## 4. Discussion

Patients hospitalized for COVID-19 with interstitial pneumonia and hypoxia requiring oxygen therapy were treated according to the hospital protocol for COVID-19 active during the early phase of the pandemic, including the best available supportive care treatment. The administration of high doses of calcifediol early during hospital admission significantly reduced the risk of death, with an OR of 0.47 (95% CI, 0.23–0.45) [28]. In the multivariate analysis, the odds ratio remained practically constant (0.476), although it did not reach statistical significance (*p* = 0.053). However, we consider that this slight discrepancy in the results between the multivariate and univariate analyses does not weaken the clinical-biological relationship between calcifediol treatment and decreased COVID-19 mortality. This is in line with what has been reported previously in patients treated with high doses of calcifediol, either associated or not with corticosteroids, whereby calcifediol reduced the risk of death and/or improved the prognosis [28,33,34]. When, as in previously reported studies, calcifediol is administered at low doses, there is no significant reduction in the mortality risk [35].

Observational studies reported the existence of an association between patients’ deficient 25(OH)D status and an increased risk of suffering from COVID-19, or greater severity and mortality from the disease [36,37,38]. In Spain, despite long hours of sunlight, low 25(OH)D serum levels are highly prevalent in the general population [39] and are even lower in patients admitted for COVID-19 [40]. On admission, hospitalized patients whose 25(OH)D levels were measured had marked 25(OH)D deficiency (overall median of 17.5 ng/mL). In patients who had not been prescribed vitamin D or calcifediol treatment prior to admission, a more marked 25(OH)D deficiency was observed compared to those who had been prescribed it (15.2 vs. 22.2 ng/mL; *p* < 0.001).

To explain the decreased 25(OH)D serum levels in COVID-19 patients, in addition to deficiency observed in the general population, so-called reverse causality cannot be ruled out. The collision effect defies association studies. Reverse causality is one of them in COVID-19 disease [41,42]. Indeed, systemic inflammation, already from several days before admission through various mechanisms [42], constitutes a significant contributing factor to the decreased serum 25(OH)D levels reported in patients with COVID-19 [43]. In any case, 25(OH)D deficiency, regardless of its cause, conditions the lack of availability of substrate for the synthesis of 1,25(OH)_2_D, which acts on the VDR. This results in the loss of the potential protective actions of the endocrine system of vitamin D in COVID-19 and constitutes a negative factor in the prognostic evolution of the disease [44].

In patients treated with vitamin D_3_ and/or calcifediol before admission, the resulting higher 25(OH)D serum levels did not lead to better severity parameters at admission. However, in the in-hospital calcifediol treatment group, more patients were pre-treated with calcifediol or vitamin D_3_. Patients not pre-treated prior to admission with vitamin D_3_ and/or calcifediol and not treated during admission with calcifediol had a higher percentage of death, but our study was not powered to detect a significant difference. This could support previous data suggesting that prescriptions of calcifediol or cholecalciferol established prior to hospitalization are associated with better survival rates among patients hospitalized with COVID-19. This finding agrees with several previously reported observational studies that have highlighted the protective effect on mortality risk in patients treated prior to admission for COVID-19 with calcifediol and/or native vitamin D for other health goals, such as osteoporosis [45,46].

Thirty-four patients (23% of the 148 with 25(OH)D levels and 14.8% of the total) had severe 25(OH)D deficiency (≤10 ng/mL) on admission. These patients had elevated serum levels of the pro-inflammatory cytokine interleukin-6 (IL-6), a prognostic indicator of mortality in COVID-19 patients [47]. The administration of calcifediol associated with corticosteroids in patients with COVID-19 significantly reduced circulating IL-6 (unpublished personal observation). In our patients, the administration of high doses of calcifediol associated with corticosteroids during hospital treatment significantly decreased the risk of death [0/16 (0%) versus 7/18 (38.9%), OR = 0.05, *p* = 0.008].

We used calcifediol instead of native vitamin D_3_ (cholecalciferol) as a therapeutic strategy because of its pharmacokinetic advantages [48,49] that give it functional superiority for use in COVID-19. It is more hydrophilic and is absorbed through the portal venous system, and does not require hydroxylation at position twenty-five, which can be impaired during inflammatory processes and other conditions [50]. With cholecalciferol, it takes approximately more than two weeks to reach a steady-state concentration of 25(OH)D, while calcifediol is available, in stable form, in high concentrations in a few hours serve as the substrate for the synthesis of 1,25(OH)_2_D in both the kidney and other target organs in COVID-19, for its endocrine, paracrine and autocrine actions. The rapid increase in serum 25(OH)D concentrations was associated with a decrease in markers of innate immunity, including eotaxin, interleukin-12, monocyte chemoattractant protein-1 and macrophage inflammatory protein-1 beta [49].

At the beginning of COVID-19, SARS-CoV-2 could modify the host’s innate immunity by deregulating type I interferon (IFN) immune responses [51]. Several of its proteins inhibit RIG-1 and MDA-5 and the activation of interferon regulatory factors (IRF3 or IRF7) necessary to produce IFN-α/β cytokines [52]. The availability of 25(OH)D could lead to upregulation of RIG-1, MDA5 and IRF3 in SARS-CoV-2 infection [53]. This action is key and explains one of the mechanisms by which calcifediol can improve innate immunity and, therefore, the survival of patients with severe COVID-19 and other viral infections [54]. In addition, activation of the VDR can promote positive effects against COVID-19, improving the antiviral response by boosting the production of antimicrobial peptides such as cathelicidin (cAMP/LL37) and β-defensin (DEFB4), as well as the modulation and induction of viral autophagy [55]. VDR activation exerts an antioxidant effect on monocytes by upregulating glutathione reductase and glutamate-cysteine ligase, reducing the production of oxygen radicals. Furthermore, VDR activation contributes to maintaining the integrity of the pulmonary epithelial barrier and stimulating its repair [38].

Moreover, the autocrine signaling of the VDES deactivates pro-inflammatory programs through VDR activation, which inhibits antigen presentation by dendritic cells, reduces pro-inflammatory T helper 1 (Th1) cells and promotes the transition to Th2 and T-regulatory (Treg) cells, inducing a shift from an inflammatory state to a more tolerogenic one [56]. This moderates the intensity of the local and systemic inflammatory immune response, which conditions the change from a severe to a more favorable clinical course of COVID-19 [38].

Another VDES mechanism that contributes to reducing the severity of COVID-19 is the powerful negative regulation of the RAAS, by inhibiting the angiotensin-converting enzyme 1 (ACE1)/angiotensin II/AT1R cascade and inducing the ACE2-enhancing effect of angiotensin (1–7) on its receptor (MasR). This promotes systemic anti-inflammatory, anti-fibrotic, antioxidant and anti-apoptotic pathways, and reduces vasoconstriction and thrombogenesis, which contributes to reducing the severity of all aspects of ARDS, post-COVID fibrosis [38], and probably those related to prolonged COVID-19 [57].

Virtually all the patients were being treated with corticosteroids. Corticosteroids are widely used to treat a range of medical conditions due to their ability to suppress the immune system and reduce inflammation by inhibiting the expression of multiple pro-inflammatory cytokines/chemokines, as well as modifying the activity of different immune cells in a way that resembles VDR stimulation in VDES. Stimulation of VDR and corticosteroid receptors has a very similar action profile, so the combined use in patients with COVID-19 may modify the contribution of each individually [56]. The association of calcifediol with corticosteroids decreased the risk of death. In a powerful observational macro-study, also during the first waves of COVID-19 infection, involving 26,508 veterans and reviewing the interaction of ‘vitamin D’ (including vitamin D2, D3, and calcitriol) and corticosteroids, the use of ‘vitamin D’ alone or in association with corticosteroids decreased the risk of death in relation to the use of corticosteroids alone, in line with our results [58]. However, corticosteroids impair the production of antiviral cytokines (IFN I) and their signaling pathway, causing a decrease and delay in the expression of IFN-stimulated genes [59]. Therefore, if corticosteroids are administered at the beginning of viral infection, they may interfere with and reduce the effectiveness of IFN production and the downregulation of IFN-stimulated genes [60], facilitating viral replication and propagation by enhancing the mechanisms of action of SARS-2 on innate immunity [21]. Corticosteroids also downregulate the mRNA expression of the antimicrobial peptide genes cAMP and β-DEFB4, lysozyme (LZY), and secretory leukocyte proteinase inhibitor 1 (SLPI) in vitro and in vivo, and reduce the expression of the human cathelicidin gene enhanced by VDR stimulation [61]. Furthermore, corticosteroids upregulate multiple components of RAAS in their ACE1 pathway, downregulating ACE2, which contributes to perpetuating inflammation [62].

Therefore, corticosteroids are effective in reducing the maladaptive hyperinflammatory response, but by decreasing innate immunity, they could enhance the evasive immune effect of SARS-2, an action that is especially serious in the most susceptible patients, decreasing viral clearance [19,21]. This dual effect would explain the paradox of improved or worsened prognosis, even death, depending on the timing of corticosteroid administration [63]. Among hospitalized patients who received corticosteroids (e.g., dexamethasone) in our study, the use of calcifediol was associated with fewer deaths [33].

The use of combined therapy (corticosteroids and baricitinib) in seriously ill patients with COVID-19 has also been reported, according to the results of the RECOVERY, COV-BARRIER, and other trials [64,65]. In our observational study, we found that the combination of corticosteroids and baricitinib did not improve mortality rates compared to corticosteroid monotherapy, as recently shown by an observational study using a Japanese multicenter database of patients hospitalized with COVID-19, which included 7433 patients in a combination treatment group (n = 679) and a control group (n = 6754). The combination of baricitinib and corticosteroid therapy did not improve mortality rates compared to corticosteroid monotherapy, probably due to the overlapping effects of both drugs [66].

When calcifediol was added to the combination of corticosteroids and baricitinib, mortality results improved, although to a lesser extent (not reaching statistical significance in our study) than when calcifediol was added to corticosteroids alone, as we have observed in the association between corticosteroids and calcifediol in this and other studies, probably due to the overlapping effects of both drugs.

The main limitation of our study is that it is not a randomized clinical trial. It also has other limitations. One of these could be a residual confounding factor or inadequate control of other factors explaining the difference in mortality, although we included the relevant clinical conditions that could be retrieved from the medical records, using a retrospective methodology. We used the Charlson Comorbidity Index (CCI) to control for comorbidity [67], in addition to individual clinical conditions, to show a measure of comorbidity as a confounding risk factor for mortality. We found no significant differences between the treatment and non-treatment groups.

This retrospective observational cohort study, which included consecutive patients hospitalized for COVID-19 and treated with corticosteroids in almost the entire cohort and baricitinib in some patients, according to clinical practice guidelines active at the time of the present study, and high doses of calcifediol in others, allows us to evaluate the effect of calcifediol on the risk of death. Patients treated with calcifediol had a lower risk of death than those treated with corticosteroids alone. Treatment with baricitinib in those treated with corticosteroids did not improve the risk of death, which did, however, improve when calcifediol was added to this combination (not significantly).

## 5. Conclusions

Based on all the available accumulated evidence and the data from the present study, we can conclude that calcifediol may represent a promising adjunctive treatment in hospitalized patients with COVID-19, but randomized controlled trials are needed to confirm its efficacy and establish it as a standard treatment option. On the other hand, a recommendation can be made not to administer corticosteroids or other anti-inflammatory drugs such as baricitinib until the natural history of the disease reaches a stage of severe hyperinflammation.

Calcifediol is an inexpensive treatment, with no significant adverse effects, with antiviral action, which reduces the reactive hyperinflammatory response, and can even be used in an early phase of the disease. This could have positive implications for the treatment of severe COVID-19 requiring hospitalization and other viral diseases that present with cytokine/chemokine crises, due to its actions on innate and acquired immunity [27]. The retrospective observational design and sample size limit the interpretation of these findings. To properly validate our observations, the results of large-scale randomized controlled trials with calcifediol would be required.

## Figures and Tables

**Table 1:** Characteristics of the patients, on admission, according to the treatment group.

Characteristic	Calcifediol (N = 119, 51.7%)	Non-Calcifediol (N = 111, 48.3%)	*p*-Value
Age—years	72.9 ± 14.2	74.0 ± 16.1	0.358
Sex, males—*n* (%)	63 (52.9)	62 (55.9)	0.692
Hypertension—*n* (%)	85 (71.4)	70 (63.1)	0.206
Diabetes mellitus—*n* (%)	34 (28.6)	31 (29.9)	1.000
Obesity—*n* (%)	30 (25.2)	22 (19.8)	0.348
Cardiovascular disease—*n* (%) ^δ^	31 (26.1)	23 (20.7)	0.355
COPD/Asthma—*n* (%)	18 (15.1)	14 (12.6)	0.704
Charlson Index—*n* (%)			
0	48 (43.2%)	43 (38.7%)	0.428
1	30 (27.0%)	20 (18.0%)	
2	12 (10.8%)	21 (18.9%)	
3	13 (11.7%)	8 (7.2%)	
4–8	15 (12.6%)	18 (16.2%)	
Corticosteroids—*n* (%)	119 (100.0)	108 (97.2)	0.111
Baricitinib—*n* (%)	81 (61.8)	50 (38.2)	0.001
Previous vitamin D or calcifediol treatment—n (%)	45 (37.8)	20 (18.0)	0.001
25(OH)D (ng/mL)—Median (IQR) ^₴^	17.2 (13.5)	17.8 (15.6)	0.761
SpO_2_ (%)	89.4 ± 5.5	90.2 ± 4.6	0.308
SpO_2_/FiO_2_	319.3 ± 88.6	330.9 ± 90.7	0.274
Lymphocytes(1000/mL)	0.93 ± 0.48	1.07 ± 1.05	0.469
CRP (mg/dL)	101.1 ± 77.0	106.7 ± 75.5	0.496
Creatinine (mg/dL)	1.08 ± 0.44	1.36 ± 1.34	0.288
LDH (U/L)	312.4 ± 117.7	314.4 ± 126.1	0.876
Ferritin (ng/mL)—Median (IQR)	446 (610)	603 (1003)	0.022
IL6 (pg/mL)—Median (IQR) ^&^	45.8 (75.6)	53.4 (78.4)	0.746
D-dimer (ng/mL)—Median (IQR)	851.5 (734)	1093 (1725.5)	0.008

Plus-minus values are means ± SD. ^δ^ Includes heart failure, ischemic heart disease, cerebrovascular disease, and peripheral arterial ischemia; ^₴^ *n* = 82 (68.9%), treated; *n* = 66 (59.5%), not treated; ^&^ *n* = 82 (68.9%) treated; 81 (73.0%), not treated. Abbreviations: CRP, c-reactive protein; COPD, chronic obstructive pulmonary disease; IL6, interleukin 6; LDH, lactate dehydrogenase; SpO_2_, peripheral arterial oxygen saturation; FiO_2_, inspired fraction of oxygen.

**Table 2:** 25(OH)D levels and parameters of severity at admission and mortality according to previous treatment with or without vitamin D_3_ or calcifediol.

Characteristic	Previous Treatment (N = 65, 28.3%)	No Previous Treatment (N = 165, 71.7%)	*p*-Value
Age—years	76.2 ± 13.2	72.3 ± 15.7	0.118
Sex, males—*n* (%)	30 (46.2%)	95 (57.6%)	0.142
25 (OH)D (ng/mL)—Median (IQR) ^δ^	22.2 (18.9)	15.2 (13.1)	<0.001
SpO_2_ (%)	90.1 ± 4.5	89.6 ± 5.3	0.584
SpO_2_/FiO_2_	320.7 ± 94.6	326.5 ± 87.8	0.608
Lymphocytes (1000/mL)	0.95 ± 0.43	1.01 ± 0.92	0.668
CRP (mg/dL)	102.5 ± 76.8	104.1 ± 76.1	0.786
Creatinine (mg/dL)	1.20 ± 0.52	1.23 ± 1.13	0.223
LDH (U/L)	297.1 ± 115.5	319.8 ± 123.7	0.158
Ferritin (ng/mL)—Median (IQR)	446 (682)	575 (894)	0.194
IL6 (pg/mL)—Median (IQR) ^&^	42.7 (57.1)	52.6 (82.1)	0.518
D-dimer (ng/mL)—Median (IQR)	915.5 (1045.5) 8	971 (960.5)	0.789
Mortality (%) [95% CI]	8 (12.3%) [6.4–22.5]	33 (20.0%) [14.6–26.8]	0.187

Plus-minus values are means ± SD. ^δ^ *n* = 41 (63.1%), treated; *n* = 107 (64.8%), not treated; ^&^ *n* = 45 (69.2), treated; *n* = 118 (71.5%), not treated. Abbreviations: CI, confidence interval; CRP, c-reactive protein; IL6, interleukin 6; LDH, lactate dehydrogenase; SpO_2_, peripheral arterial oxygen saturation; FiO_2_, inspired fraction of oxygen.

**Table 3:** Characteristics of patients with severe vitamin D deficiency at admission (≤10 ng/mL), compared with patients without severe vitamin D deficiency at admission (>10 ng/mL).

Characteristic	Severe vit D Deficiency (N = 34, 14.8%)	No Severe vit D Deficiency (N = 114, 49.6%)	*p*-Value
Age—years	78.0 ± 13.7	71.6 ± 14.5	0.012
Sex, males—*n* (%)	17 (50.0%)	67 (58.8%)	0.432
Mortality—*n* (%)	7 (20.6%)	19 (16.7%)	0.612
Hypertension—*n* (%)	26 (65.8%)	75 (65.8%)	0.297
Diabetes—*n* (%)	12 (35.3%)	30 (26.3%)	0.386
Obesity—*n* (%)	3 (8.8%)	20 (26.3%)	0.035
Cardiovascular disease—*n* (%)	9 (26.5%)	28 (24.6%)	0.824
COPD/Asthma—*n* (%)	3 (8.8%)	15 (13.2%)	0.765
Corticosteroids—*n* (%)	32 (94.1%)	114 (100%)	0.052
Baricitinib—*n* (%)	16 (47.1%)	74 (65.5%)	0.071
Previous vitamin D_3_ or calcifediol treatment	4 (11.8%)	37 (32.5%)	0.017
SpO_2_ (%)	89.5 ± 4.5	90.0 ± 4.1	0.095
SpO_2_/FiO_2_	306.5 ± 89.3	331.6 ± 91.7	0.117
Lymphocytes (1000/mL)	1.03 ± 0.81	1.0 ± 0.96	0.834
CRP (mg/dL)	119.8 ± 93.4	103.9 ± 71.1	0.545
Creatinine (mg/dL)	1.23 ± 0.66	1.10 ± 0.49	0.309
LDH (U/L)	299.7 ± 135.9	326.1 ± 130.8	0.149
Ferritin (ng/mL)—Median (IQR)	450 (1111	541 (728)	0.826
IL6 (pg/mL)—Median (IQR) ^&^	76.9 (84.4)	40.7 (68.5)	0.019
D-dimer (ng/mL)—Median (IQR)	965.5 (1682.8)	910.0 (849.0)	0.082

Plus-minus values are means ± SD. ^&^ includes heart failure, ischemic heart disease, cerebrovascular disease, and peripheral arterial ischemia; *n* = 41 (63.1%), treated; *n* = 107 (64.8%), not treated; *n* = 45 (69.2%), treated; *n* = 118 (71.5%), not treated. Abbreviations: CRP, c-reactive protein; IL6, interleukin 6; LDH, lactate dehydrogenase; SpO_2_, peripheral arterial oxygen saturation; FiO_2_, inspired fraction of oxygen.

**Table 4:** Mortality with or without calcifediol treatment.

	Calcifediol Mortality (*n*, %, 95%CI )	No Calcifediol Mortality (*n*, %, 95% CI)	OR (95% CI)	*p* Value
Global—*n* = 230	15/119 (12.6%, 7.8–19.8)	26/111 (23.4%, 16.5–32.1)	0.47 (0.23–0.45)	0.039
Males	8/63 (12.7%, 6.6–23.1)	18/62 (29.0%, 19.2–41.3)	0.36 (0.14–0.89)	0.029
Women	7/56 (12.5%, 6.2–23.6)	8/49 (16.3%, 8.5–29.0)	0.73 (0.24–2.19)	0.590
Severe vitamin D deficiency—*n* = 34	0/16 (0%, 0.0–19.4)	7/18 (38.9%, 20.3–61.4)	0.05 (0.002–0.90)	0.008
No previous vitamin D_3_ or calcifediol treatment—*n* = 165	10/74 (13.5%, 7.5–23.1)	23/91 (25.3%, 17.5–35.1)	0.46 (0.20–1.04)	0.078
Previous vitamin D_3_ or calcifediol treatment—*n* = 65	5/45 (11.1%, 4.8–23.5)	3/20 (15.0%, 5.2–36.0)	0.71 (0.15–3.3)	0.693

**Table 5:** Mortality according to treatment among the different groups, according to the use of corticosteroids, calcifediol and baricitinib.

	Corticosteroids (N = 60, 61.2%)	Corticosteroids and Calcifediol (N = 38, 38.8%)	Corticosteroids and Baricitinib (N = 50, 38.2%)	Corticosteroids and Calcifediol and Baricitinib (N = 81, 61.8%)	*p* Value
Mortality (%) [95%] CI	14 (23.3%) [14.4–35.4]	1 (2.6%) [0.5–13.5]	12 (24.0%) [14–37.4]	14 (17.3%) [10.6–26.9]	0.018
Age—years	73.8 ± 18.4	72.2 ± 15.1	74.4 ± 13.3	73.1 ± 13.8	0.922
Serum 25(OH)D levels—mean	N = 29 17.0 ± 10.6	N = 28 20.0 ± 15.2	N = 36 24.2 ± 20.0	N = 54 20.8 ± 12.3	0.285
Previous vitamin D_3_ or calcifediol treatment (%)	11 (18.3%)	19 (50.0%)	9 (18.0%)	26 (32.1%)	0.002

## Data Availability

Some or all of the datasets generated during and/or analyzed during the current study are not publicly available due to privacy but are available from the corresponding author on reasonable request.

## Supplementary Materials

The following are available online at https://www.mdpi.com/article/10.3390/nu17121968/s1, Table S1: Descriptive analysis of the group excluded due to inadequate treatment with calcifediol. Table S2: Univariate analysis in the total population. Table S3: Multivariate analysis in the total population. Table S4: Univariate analysis in males. Table S5: Multivariate analysis in males. Table S6: Univariate analysis in patients with severe vitamin D deficiency.

## Appendix A

### Collaborators in Data Collection

Teresa Granero-Salas, Andrea Pérez-Trujillo, Marta Guzman-Pérez, Marcos-Alexander Ostaiza-Ordoñez, Rocio Garvi-Merino, Jordi Olucha-Puchol, Cristina Garcia-Gomez, Cristina del Pozo-Carlavilla, Belen Serna-Serrano, Juan Manuel Collado-Sanz, Hector Alabort-Ayllón, Beira da Silva-Cabañero.

From the Departments of Internal Medicine (T.G.-S., A.P.-T., M.G.-P., M.-A.O.-O., R.G.-M., J.O.-P.), Hospital Pharmacy (C.G.-G., C.P.-C., B.S.-S., J.M.C.-S., H.A.-A.) and Clinical Biochemistry (B.S.-C.). All at Complejo Hospitalario Universitario de Albacete, 02008 Albacete, Spain.
