# Supplementary material for: ALBACOVIDIOL Study: Effect of Calcifediol Treatment on Mortality in Patients Hospitalized for COVID-19: A Retrospective Analysis
Source: Nutrients. 2025 Jun 10;17(12):1968. doi: 10.3390/nu17121968 (PMC12196532; doi:10.3390/nu17121968)
Supplement: Supplementary file 1 [file nutrients-17-01968-s001.zip › nutrients-3624558-supplementary.pdf]

Supplementary tables

Table S1. Descriptive analysis of the group excluded due to inadequate treatment with calcifediol.

**Characteristic**	**All patients (N=275, 100%)**	**Included (N=230, 83.6%)**	**Excluded (N=45 16.4%)**	**P-value**
Age—years	73.3±15.5	73.4±15.1	73.0±17.2	0.827
Sex, males—n (%)	146 (53.1)	125 (54.3)	21 (46.7)	0.415
Mortality	51 (18.5)	41 (17.8)	10 (22.2)	0.529
Hypertension—no (%)	191 (69.5)	155 (67.4)	36 (80.0)	0.112
Diabetes mellitus—no (%)	83 (30.2)	65 (28.3)	18 (40.0)	0.155
Obesity—no. (%)	64 (23.3)	52 (22.6)	12 (26.7)	0.566
Cardiovascular disease—no (%)	68 (24.7)	54 (23.5)	14 (31.1)	0.344
COPD/Asthma—no (%)	40 (14.5)	32 (13.9)	8 (17.8)	0.492
Charlson Index---(%)				
0	107 (39.3)	91 (39.9)	16 (36.4)	0.163
1	60 (22.1)	50 (21.9)	10 (22.7)	
2	41 (15.1)	33 (14.5)	8 (18.2)	
3	23 (8.5)	21 (9.2)	2 (4.5)	
4-8	41 (15.1)	33 (14.5)	8 (18.2)	
Corticosteroids—n (%)	271 (98.5)	227 (98.7)	44 (97.8)	0.513
Baricitinib—no. (%)	153 (56.2)	131 (57.2)	22 (51.2)	0.505
Previous vitamin D treatment—n (%)	81 (29.5)	65 (28.3)	16 (35.6)	0.372
25 (OH)D (ng/mL)—Median (IQR)₴	20.8 (15.0)	20.7 (14.7)	21.5 (17.2)	1.000
SpO2 (%)	89.4±5.6	89.8±5.1	87.7±7.7	0.095
SpO2/FiO2	321.4±91.8	324.9±89.6	304.0±101.5	0.134
Lymphocytes(1000/mL)	0.99±0.76	1.00±0.81	0.98±0.46	0.456
CRP (mg/dl)	103.6±77.6	103.7±76.1	103.3±85.9	0.651
Creatinine (mg/dL)	1.21±0.94	1.22±1.00	1.19±0.62	0.800
LDH (U/L)	318.3±134.8	313.3±121.6	343.5±188.3	0.677
Ferritin (ng/m)	775.6±851.4	803.5±857.8	628.0±810.4	0.087
IL6 (pg/ml)	47.2 (76.2)	47.5 (76.6)	42.2 (71.0)	0.977
D-dimer (ng/ml)	934.5 (900.2)	941.0 (1006.5)	894.0 (761.0)	0.270

Plus-minus values are means ± SD. includes heart failure, ischemic heart disease, cerebrovascular disease, and peripheral arterial ischemia; ₴ n=82 (68.9%), treat; n=66 (59.5%), no treat; n=82 (68.9%) treat; 81 (73.0%), no treat. Abbreviations: CRP, c-reactive protein; COPD, chronic obstructive pulmonary disease; IL6, interleukin 6; LDH, lactate dehydrogenase; SpO2, peripheral arterial oxygen saturation; FiO2, inspired fraction of oxygen

**Table S2**. Univariate analysis in the total population		
n=230		
**Variable**	**OR [CI 95%]**	**p-value**
Calcifediol treatment	0.479 [0.236 – 0.943]	**0.033***
Age	1.09 [1.05 – 1.14]	**<0.001***
Sex (female)	0.64 [0.32 – 1.27]	0.204
Hypertension	1.36 [0.66 – 2.96]	0.406
Diabetes mellitus	1.24 [0.59 – 2.516]	0.561
Obesity	1.56 [0.72 – 3.25]	0.249
Cardiovascular disease	1.95 [0.93 – 3.98]	0.078
COPD/Asthma	1.72 [0.69 – 3.95]	0.236
Previous Vitamin D_3 or calcifediol treatment	0.59 [0.25 – 1.27]	0.181
Baricitinib treatment	1.35 [0.69 - 2.75]	0.386
SpO2/FiO2	0.993 [0.989 – 0.997]	**<0.001***

Abbreviations: COPD, chronic obstructive pulmonary disease; SpO2, peripheral arterial oxygen saturation; FiO2, inspired fraction of oxygen

**Table S3**. Multivariate analysis in the total population		
n=230		
**Variable**	**OR [CI 95%]**	**p-value**
Calcifediol treatment	0.476 [0.218 – 1.010]	0.053
Age	1.081 [1.045 – 1.126]	**<0.001***
SpO2/FiO2	0.993 [0.989 – 0.997]	**0.001***

Abbreviations: SpO2, peripheral arterial oxygen saturation; FiO2, inspired fraction of oxygen

**Table S4**. Univariate analysis in males		
n=125		
**Variable**	**OR [CI 95%]**	**p-value**
Calcifediol treatment	0.37 [0.14 – 0.89]	**0.025***
Age	1.09 [1.05 – 1.15]	**<0.001***
Hypertension	1.37 [0.55 – 3.69]	0.505
Diabetes mellitus	1.68 [0.68 – 4.06]	0.260
Obesity	1.16 [0.40 – 3.05]	0.776
Cardiovascular disease	1.12 [0.41 – 2.84]	0.812
COPD/Asthma	2.10 [0.74 – 5.61]	0.158
Previous Vitamin D_3 or calcifediol treatment	1.25 [0.46 – 3.19]	0.650
Baricitinib treatment	0.96 [0.40- 2.35]	0.924
SpO2/FiO2	0.995 [0.990 – 1.000]	**0.034***

Abbreviations: COPD, chronic obstructive pulmonary disease; SpO2, peripheral arterial oxygen saturation; FiO2, inspired fraction of oxygen

**Table S5**. Multivariate analysis in males		
n=125		
**Variable**	**OR [CI 95%]**	**p-value**
Calcifediol treatment	0.23 [0.08 – 0.63]	**0.004**
Age	1.09 [1.05 – 1.15]	**<0.001***
SpO2/FiO2	0.994 [0.988 – 1.000]	**0.003***

Abbreviations: SpO2, peripheral arterial oxygen saturation; FiO2, inspired fraction of oxygen

**Table S6.** Univariate analysis in patients with severe vitamin D deficiency		
n=34		
**Variable**	**OR [CI 95%]**	**p-value**
Calcifediol treatment	0.046 [<0.001 – 0.447]	**0.004***
Age	1.06 [0.98 – 1.18]	0.144
Sex (female)	0.72 [0.14 – 3.55]	0.688
Hypertension	1.59 [0.26 – 17.06]	0.636
Diabetes mellitus	0.33 [0.03 – 1.90]	0.228
Obesity	2.35 [0.19 – 21.15]	0.460
Cardiovascular disease	1.24 [0.19 – 6.59]	0.805
COPD/Asthma	2.35 [0.19 – 21.15]	0.460
Previous vitamin D_3 or calcifediol treatment	0.348 [0.003 – 3.91]	0.445
Baricitinib treatment	0.42 [0.07- 2.11]	0.299
SpO2/FiO2	0.996 [0.987 – 1.005]	0.417

Abbreviations: COPD, chronic obstructive pulmonary disease; SpO2, peripheral arterial oxygen saturation; FiO2, inspired fraction of oxygen
